# Severe Japanese Spotted Fever With Disseminated Intravascular Coagulation and a Fatal Outcome: A Case Series of Five Patients

**DOI:** 10.7759/cureus.96389

**Published:** 2025-11-08

**Authors:** Shinsei Ota, Tetsuya Kawahara

**Affiliations:** 1 Internal Medicine, Shinkomonji Hospital, Kitakyushu, JPN; 2 Endocrinology and Metabolism, Shinkomonji Hospital, Kitakyushu, JPN

**Keywords:** creatine kinase (ck), disseminated intravascular coagulation (dic), japanese spotted fever, kruskal-wallis test, rickettsia japonica, white blood cell counts

## Abstract

Japanese spotted fever (JSF), caused by *Rickettsia japonica*, is an endemic tick-borne disease in Japan that usually responds well to tetracycline therapy. However, delayed diagnosis and treatment can result in serious complications such as disseminated intravascular coagulation (DIC) and death. To identify early markers of severe disease, a case series of five JSF patients (one male patient, four female patients; aged 61-78) treated between 2019 and 2024 was analyzed. All patients presented with fever and erythema; two exhibited eschar. Although empirical tetracycline therapy was administered in every case, initiation was delayed in three patients. Among these, two developed DIC, and one experienced multiple organ failure and died despite intensive treatment. Laboratory findings in the fatal case revealed markedly elevated white blood cell (WBC) and creatine kinase (CK) levels at presentation. Statistical analysis (Kruskal-Wallis test) demonstrated significantly higher WBC (p = 0.023) and CK (p = 0.041) values in the fatal case compared with survivors, while platelet count, C-reactive protein, procalcitonin, and liver enzyme levels showed no significant differences. These findings emphasize the importance of early suspicion and prompt antibiotic therapy for JSF, particularly when patients present with fever, rash (including palms and soles), and eschar. Delay in tetracycline initiation correlated with prolonged hospitalization and poor outcomes. Elevated WBC and CK at admission may represent early prognostic indicators for severe JSF, warranting close monitoring and aggressive management. In summary, delayed treatment and high initial WBC and CK were associated with progression to DIC and death in this series. Rapid diagnosis and immediate tetracycline therapy are essential to prevent severe complications.

## Introduction

Japanese spotted fever (JSF) is a tick-borne infectious disease caused by *Rickettsia japonica* and transmitted to humans through bites from infected ticks. Other tick-borne diseases include scrub typhus and severe fever with thrombocytopenia syndrome (SFTS). Scrub typhus is caused by *Orientia tsutsugamushi *and is also transmitted by mite bites. Although both diseases commonly present with fever, rash, and an eschar, JSF is distinguished by the frequent appearance of rash on the palms and soles. The seasonal distribution also differs: JSF typically occurs from spring to autumn, while scrub typhus tends to appear in late autumn [1].

In differentiating between JSF and SFTS, hematological findings are particularly useful, with marked differences observed in leukopenia of WBC < 4000/µL count between the two diseases. In addition, reports have suggested that the absence of rash strongly suggests SFTS [2].

JSF was first reported in Japan in 1984 [3]. Initially considered rare and regionally restricted, the number of reported cases has increased since its designation under infectious disease surveillance, exceeding 300 annual cases since 2017 [4]. Environmental changes and expanded human activity are suspected contributors to this rise [5]. The incubation period ranges from 2 to 8 days; the clinical triad of fever, rash, and eschar is characteristic. The diagnosis of JSF remains difficult due to nonspecific symptoms and the limited sensitivity of serological tests during the early phase. Currently, polymerase chain reaction (PCR) and its variants, such as real-time PCR, nested PCR, and loop-mediated isothermal amplification, are the most reliable methods for detecting *Rickettsia japonica* DNA [6].

Standard treatment is tetracycline (TC) for one to two weeks; combination therapy including fluoroquinolones has been reported for refractory cases [7].

Between 2019 and 2024, five JSF cases were diagnosed and treated at our hospital in Kitakyushu city, Fukuoka prefecture. One case resulted in death. Because tick-borne infections often present with nonspecific symptoms, diagnostic delay can lead to severe complications such as disseminated intravascular coagulation (DIC) [8]. This report describes these five cases and examines factors associated with severe disease progression. Informed consent for publication was obtained from patients, families, or related parties.

To inform future treatment strategies, we analyzed laboratory data from all five cases from the day of admission to day 3 and examined parameters that showed significant changes in patients with severe disease. Because the sample size was small and each patient (n = 5) had only three measurements (days 1-3), the data were considered non-parametric, and the Kruskal-Wallis test was used to compare differences among the groups. When a significant difference was detected across all cases, post hoc pairwise comparisons were performed using the Steel-Dwass test, with Case 1 (the severe case) serving as the reference group (Case 1 vs. 2, Case 1 vs. 3, Case 1 vs. 4, Case 1 vs. 5). Although a two-sided p-value of < 0.05 was considered statistically significant, the Bonferroni correction was applied to adjust for multiple comparisons, resulting in a significance threshold of p < 0.05 / 4 = 0.0125. All statistical analyses were performed using R software, Version 4.5.1 (R Foundation for Statistical Computing, Vienna, Austria).

All participants or their family members were provided with an explanation regarding the protection of anonymity, and consent for publication of the study was obtained.

## Case presentation

Case 1

**Figure 1:**
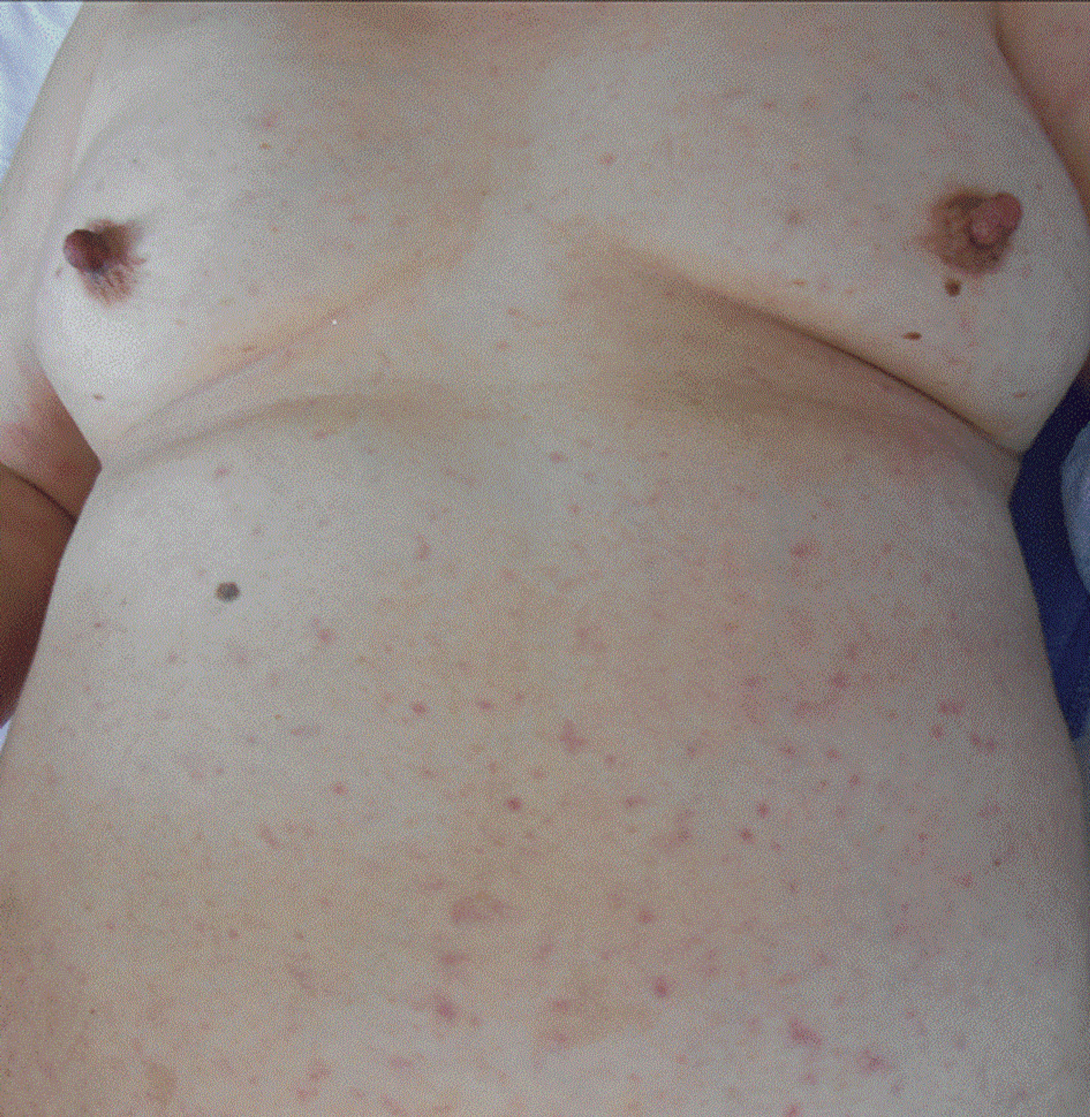
Multiple poorly demarcated erythematous macules (5-10 mm in diameter) on the trunk without fusion, heat, or pruritus.

A 78-year-old woman (Case 1) presented with impaired mobility. Her medical history included lower-extremity lymphedema and dyslipidemia. On July 4, she noticed a rash while bathing, followed by a high fever the next day. She was referred to our hospital for further evaluation. Her cohabiting sister had developed fever and a generalized rash on June 28 and had been hospitalized at the same institution. The patient frequently walked near a mountain close to her home, suggesting possible exposure to arthropod vectors. Upon admission, she was alert (JCS 0) with a blood pressure of 142/72 mmHg, pulse rate of 100 beats per minute, temperature of 38.5°C, and oxygen saturation of 95% on room air. Physical examination revealed pitting edema of the lower legs and multiple poorly demarcated erythematous macules measuring 5-10 mm in diameter distributed over the head, face, trunk, extremities, palms, and soles, without fusion, heat, or pruritus (Figure 1). The oral mucosa exhibited erythema and whitish coatings on the buccal mucosa and tongue, and no eschar was observed.

**Figure 2:**
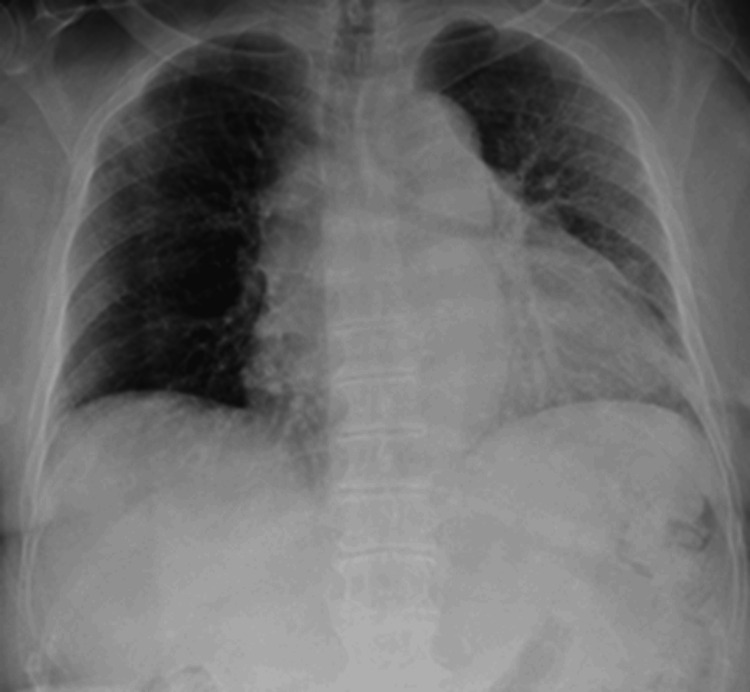
Chest X-ray in Case 1 on Day 1 from admission. Pulmonary congestion in both lungs was mild compared to that in Figure 3.

**Figure 3:**
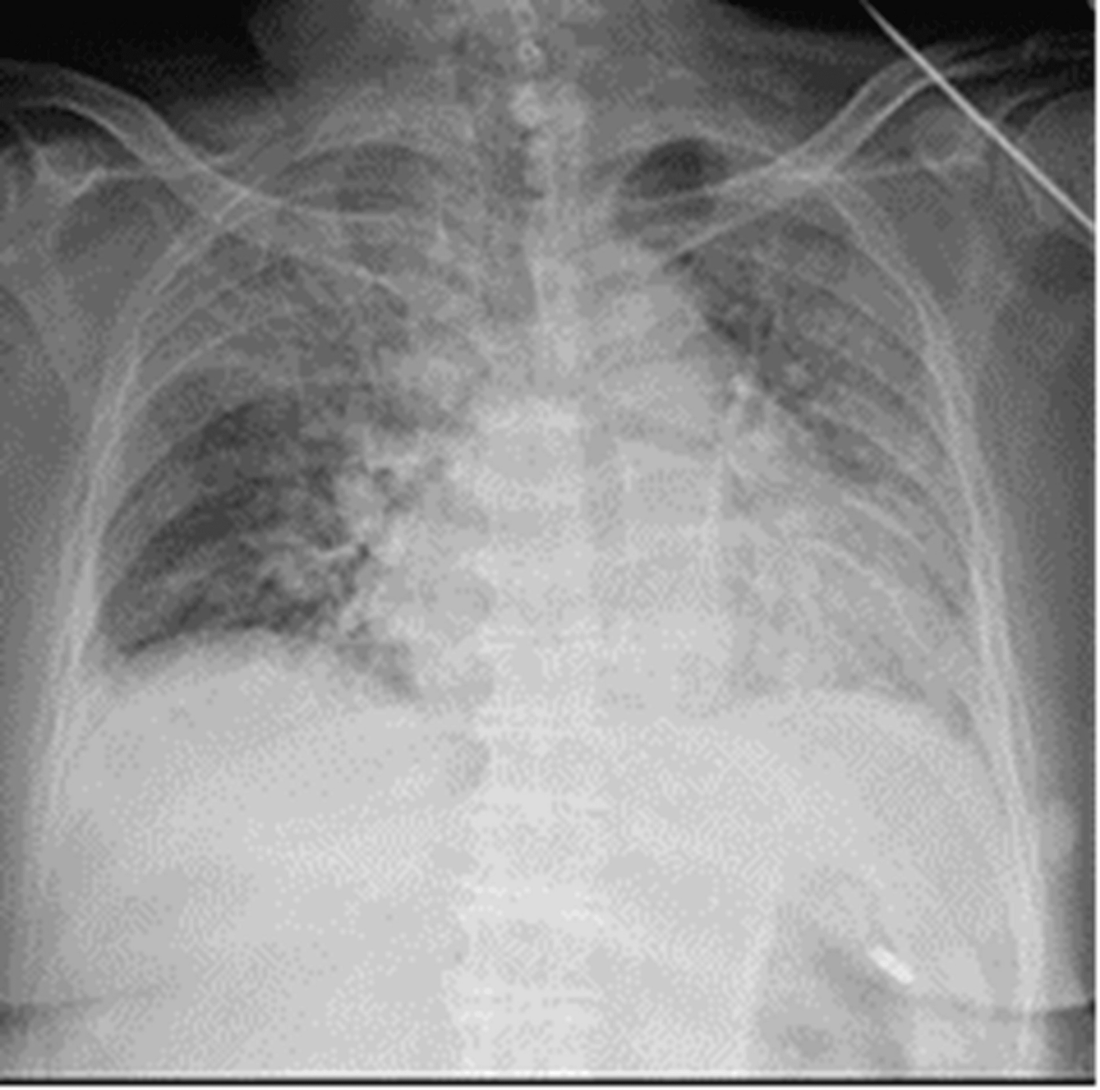
Chest X-ray in Case 1 on Day 15 from admission. Chest X-ray on the 15th day showed decreased transparency in both lungs, suggesting findings consistent with pulmonary congestion.

Initial laboratory testing showed aspartate aminotransferase (AST) 47 U/L, alanine aminotransferase (ALT) 31 U/L, sodium 136 mmol/L, potassium 3.1 mmol/L, chloride 98 mmol/L, CRP 15.03 mg/dL, and WBC 8,400/µL (neutrophils 74.0%). On July 8, she developed impaired consciousness. Laboratory findings at that time demonstrated PLT 7.7×10⁴/µL, CK 520 U/L, FDP 35.3 µg/mL, D-dimer 29.7 µg/mL, and PT-INR 1.14, fulfilling the JAAM-2 DIC diagnostic criteria (6 points) [9]. Viral serologies for rubella, measles, EBV, and CMV were negative for acute infection, as were tests for zoonoses, fungal infection, lupus nephritis, and vasculitis. Given that her sister had similar symptoms, JSF was suspected, and serum was submitted for JSF testing on July 9. Azithromycin 500 mg IV was started on July 10. Oxygen supplementation became necessary as her SpO₂ decreased to approximately 85% on room air. On July 14, minocycline 200 mg/day was initiated. A chest X-ray performed on July 15 demonstrated worsening pulmonary congestion (Figures 2, 3). Despite 5 L/min of oxygen, her SpO₂ remained around 84%, and a high-flow nasal cannula was introduced. She was intubated on July 19 and placed on mechanical ventilation; hypotension ensued, and she died on July 27. Postmortem testing of serum collected on July 22 confirmed JSF by positive IgG and IgM antibodies to *R. japonica* via indirect immunofluorescence assay. This patient developed DIC and progressive respiratory failure leading to death.

Case 2

**Figure 4:**
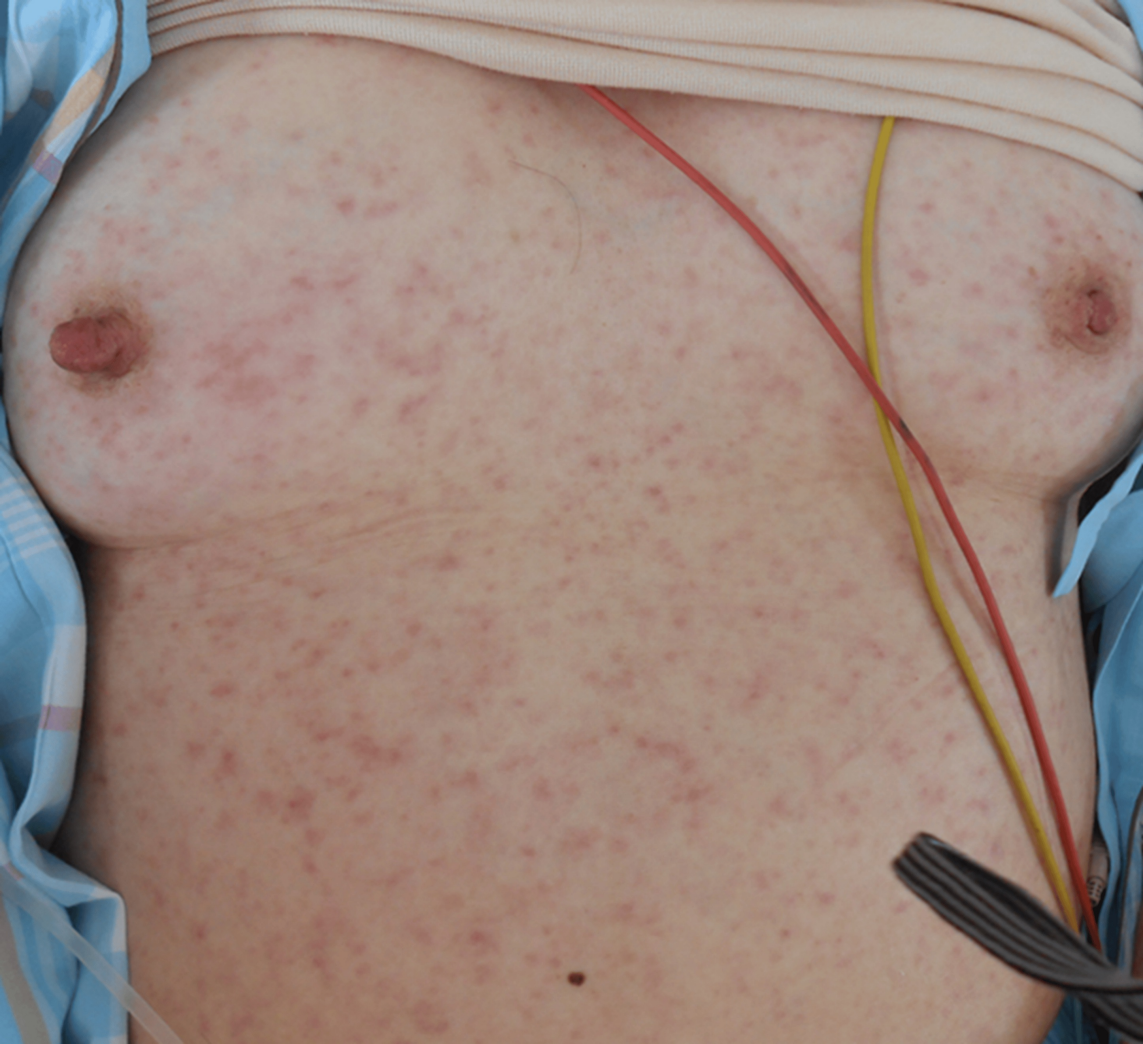
Findings of skin rashes in Case 2. Small erythema measuring 5-10 mm with indistinct borders was observed on the trunk and limbs.

A 68-year-old woman (Case 2), the younger sister and cohabitant of Case 1, presented with impaired mobility. She had developed a fever of approximately 39°C and a trunk and extremity rash on June 28. Outpatient chest X-ray findings were unremarkable, and measles/rubella IgM was negative. On July 3, thrombocytopenia and lower-leg edema were detected, prompting referral to our hospital. She also frequently walked on mountain trails near her home. Her past medical history included hypertension and anxiety disorder, for which she was treated with an angiotensin II receptor blocker and an anxiolytic. On admission, she was conscious (JCS I-3) with a blood pressure of 120/60 mmHg, pulse 76 bpm, temperature 36.9°C, and SpO₂ 96% on room air. Physical examination revealed bilateral pitting edema and poorly defined erythematous macules measuring 5-10 mm on the face, neck, extremities, and trunk (Figure 4). Laboratory studies showed AST 148 U/L, ALT 84 U/L, CK 1606 U/L, CRP 20.67 mg/dL, WBC 6,900/µL (neutrophils 83.4%), FDP 22.5 µg/mL, and thrombocytopenia.

Her initial working diagnosis included viral-associated thrombocytopenia and rhabdomyolysis, and intravenous fluids were administered. Serological tests revealed CMV IgG positive/IgM negative; ANA, β-D-glucan, MPO-ANCA, and PR3-ANCA were negative. O. tsutsugamushi antibodies (Kato, Kawasaki, Kuroki, Gilliam, Karp) were all negative. On July 9, despite persistent fever, her laboratory results showed overall improvement. Given her sister’s deterioration, intravenous azithromycin 500 mg/day was started on July 10. Fever resolved by July 13, and oral minocycline 200 mg/day was initiated due to clinical suspicion of rickettsial infection, including SFTS. By July 25, laboratory parameters had improved (AST 27 U/L, ALT 20 U/L, CRP 0.58 mg/dL, WBC 4,500/µL, PLT 36.5×10⁴/µL, CK 53 U/L), and she was discharged. Although paired sera were negative for JSF, the clinical course and epidemiological context supported a diagnosis of JSF. She recovered after delayed treatment.

Case 3

**Figure 5:**
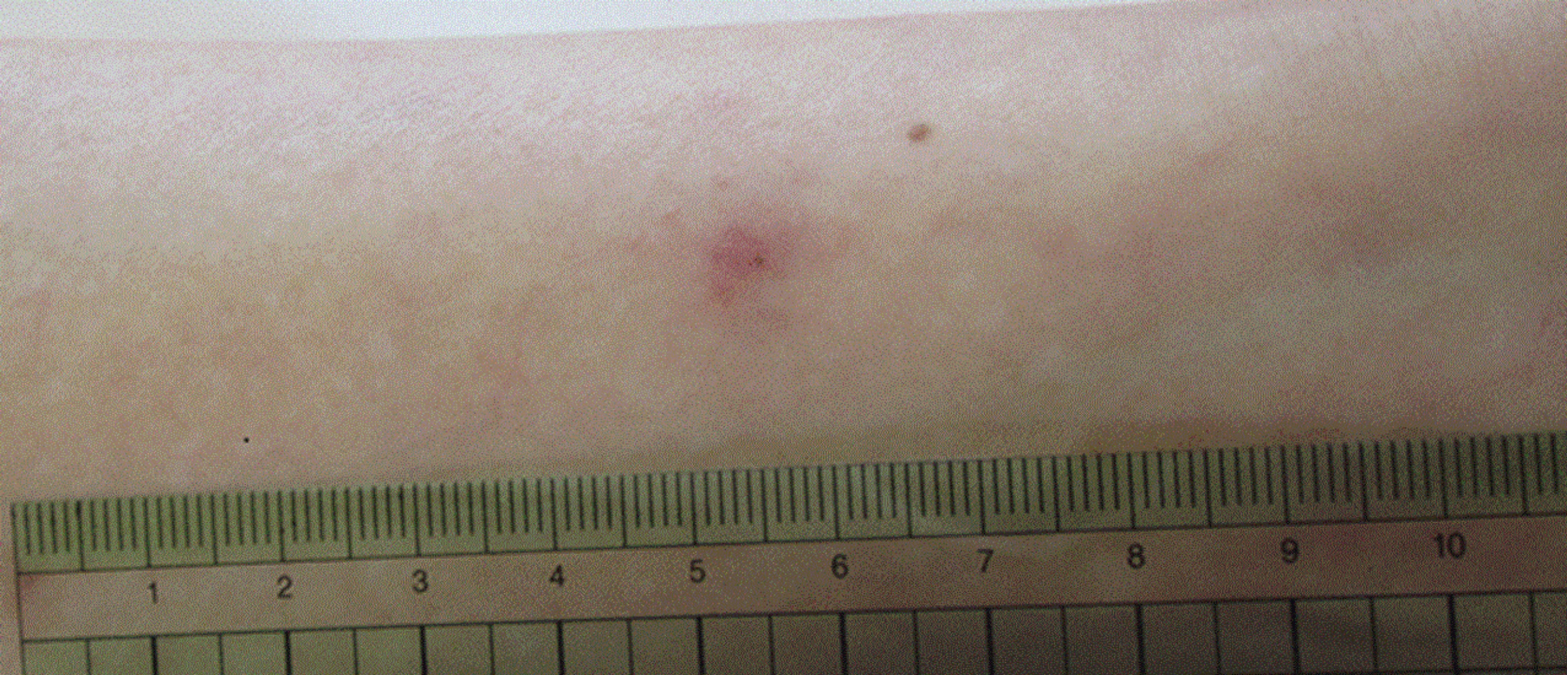
Eschar-like papule with central pigmentation on the medial aspect of the left lower leg.

A 69-year-old woman (Case 3) with no significant medical history presented with fever and malaise. Fatigue began on August 22, and she visited the emergency department on August 23 with a temperature of 38.5°C. Initial laboratory findings included AST 22 U/L, ALT 12 U/L, CRP 3.86 mg/dL, WBC 5,800/µL, and PLT 17.1×10⁴/µL. Chest X-ray findings were unremarkable. She received antipyretics and was discharged. However, fever persisted, and she returned on August 26, reporting regular walks along mountain paths. Upon readmission, she was alert (JCS 0) with blood pressure 87/51 mmHg, pulse 93 bpm, temperature 36.9°C, and SpO₂ 97% on room air. Examination revealed erythema of the left forearm and both thighs, as well as an eschar-like papule with central pigmentation on the medial aspect of the left lower leg (Figure 5). Erythema expanded to the face and neck during febrile episodes. Laboratory data on August 26 showed AST 110 U/L, ALT 63 U/L, and elevated FDP and CRP levels.

A hepatic abscess was initially suspected, and sulbactam/cefoperazone therapy was initiated. On August 27, laboratory results worsened (AST 215 U/L, ALT 125 U/L, CRP 26.2 mg/dL, WBC 2,900/µL, PLT 10.6×10⁴/µL, FDP 56.5 µg/mL, PT-INR 1.08), fulfilling the JAAM-2 DIC diagnostic criteria (score 4) [9]. Recombinant thrombomodulin was administered. MRCP ruled out hepatic abscess. Viral and autoimmune testing (CMV, EBV, measles, rubella, mumps, parvovirus B19, MPO-ANCA, PR3-ANCA) were negative. Dermatology consultation identified the eschar-like lesion and widespread erythema, raising suspicion for rickettsial infection; IV minocycline 200 mg/day was initiated on August 30. O. tsutsugamushi antibodies were negative. After two weeks of minocycline therapy, fever and rash improved. Serology performed on September 10 demonstrated positive IgG and IgM for R. japonica, confirming JSF. By September 19, laboratory results had normalized, and she was discharged. She developed DIC but survived after intensive care and tetracycline therapy.

Case 4

**Figure 6:**
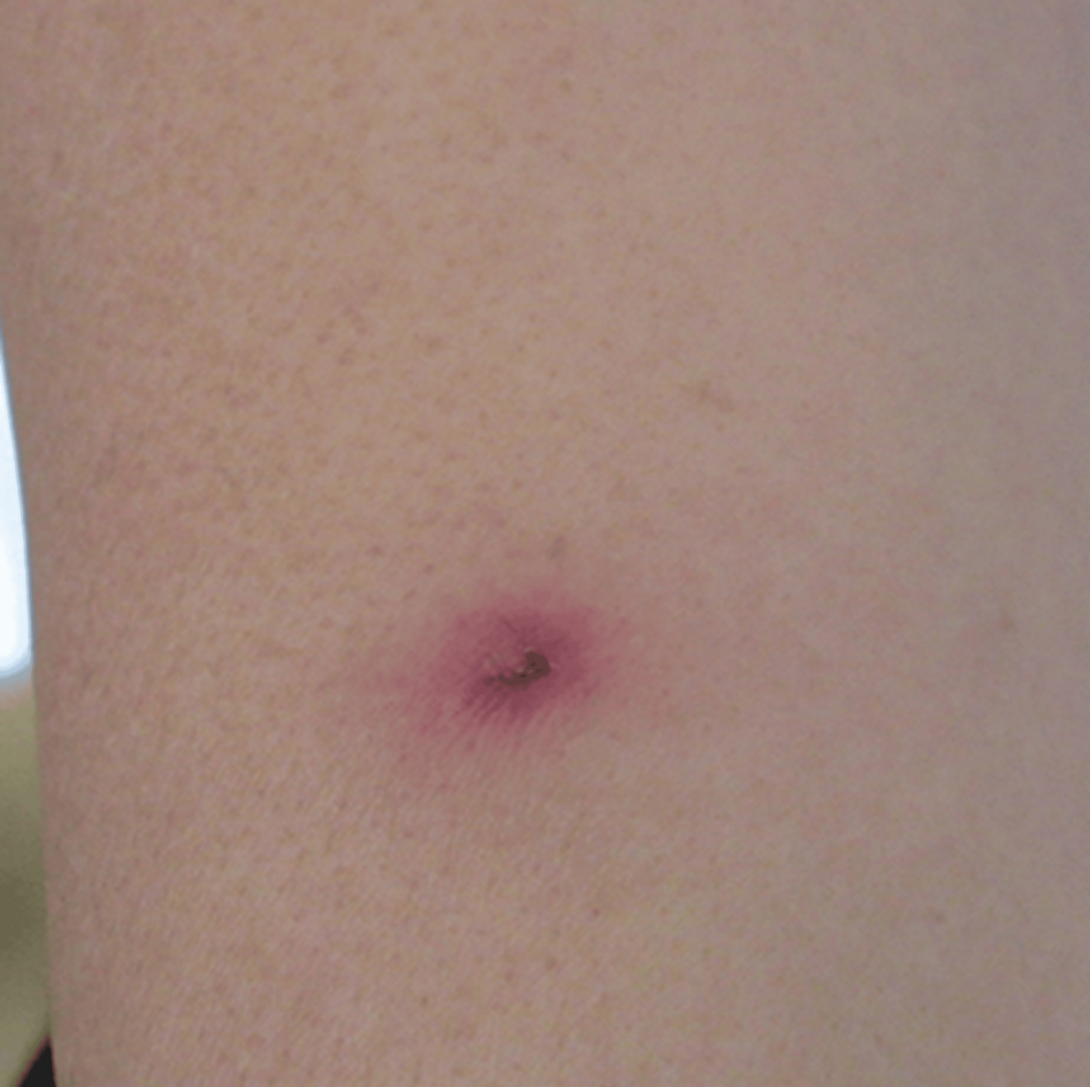
Findings of eschar in Case 4. A puncture wound was observed on the right forearm, along with a purple rash on the surrounding skin.

A 61-year-old man (Case 4) with hypertension presented after noticing a bean-sized organism fall from his right wrist while gardening on May 10, followed by fever up to 38°C. On admission, he was alert (JCS 0) with a blood pressure of 162/98 mmHg, pulse 119 bpm, temperature 38.2°C, and SpO₂ 95% on room air. Examination demonstrated a crusted lesion with surrounding erythema on the right forearm (Figure 6). Laboratory results revealed mild CRP elevation, WBC 5,200/µL (neutrophils 90.2%), PLT 16.3×10⁴/µL, and FDP 3.9 µg/mL.

Rickettsial infection was suspected, and oral tetracycline therapy was initiated. O. tsutsugamushi antibody testing was negative. On May 17, multiple nonpruritic erythematous macules appeared on the thighs and right shoulder, and by May 20, the rash had extended to the palms and soles while the fever improved. By May 31, both fever and rash had resolved, and laboratory results had normalized. Serology on June 2 demonstrated elevated JSF IgM and IgG by indirect immunofluorescence assay, confirming the diagnosis of JSF. The patient received early tetracycline therapy and recovered without complications.　

Case 5

**Figure 7:**
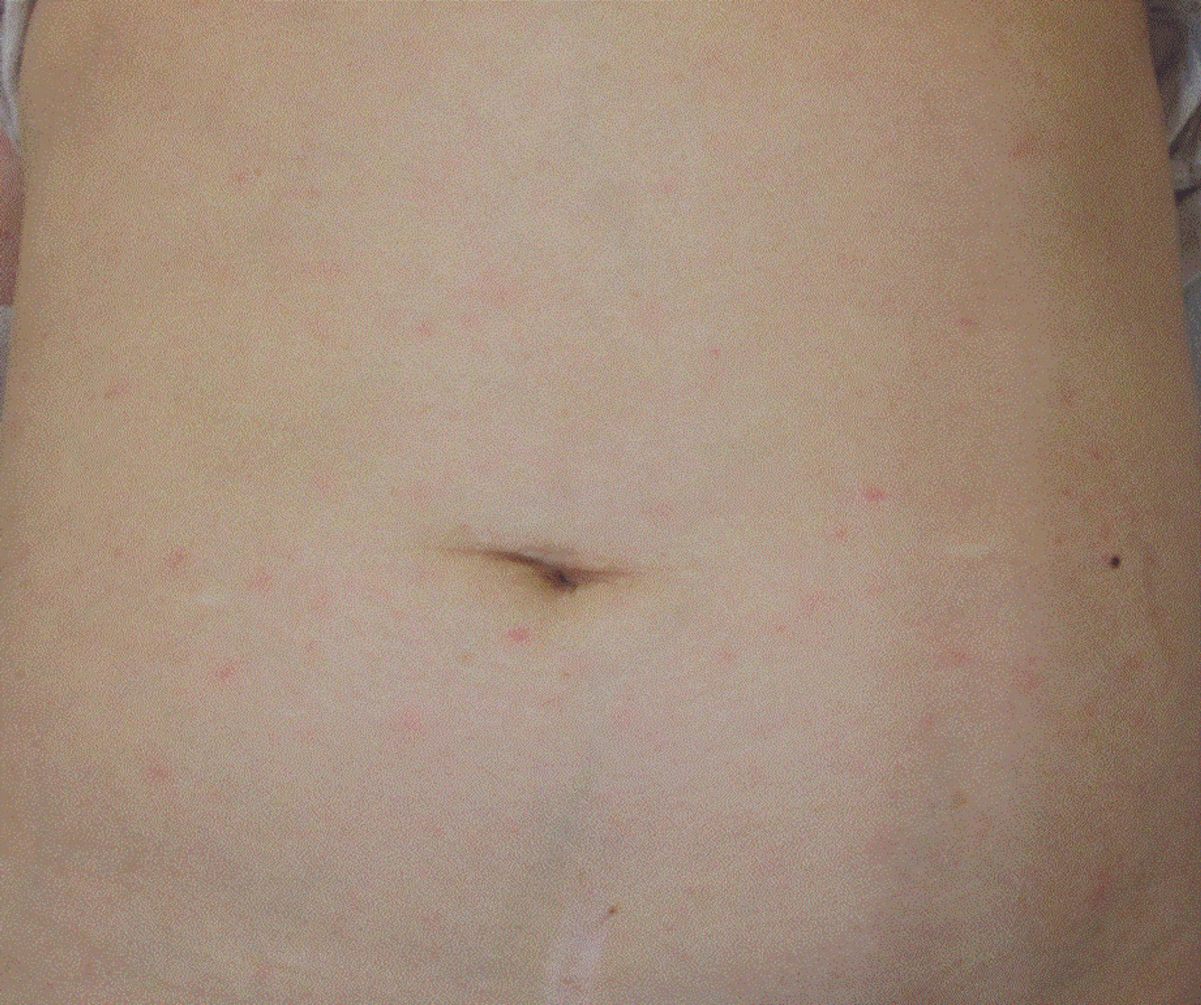
Scattered 2-3 mm erythematous macules on the trunk without eschar.

A 69-year-old woman (Case 5) noticed a rash on her right buttock on April 21 that subsequently spread systemically. On April 27, she developed headache, vomiting, and persistent fever up to 38°C. She lived in a mountainous area and suspected a tick bite. Her medical history included hypertension and colorectal cancer under outpatient follow-up. On admission, she was alert (JCS 0) with a blood pressure of 100/60 mmHg, pulse 88 bpm, temperature 36.5°C, and SpO₂ 96% on room air. Physical examination showed scattered 2-3 mm erythematous macules on the face, palms, and trunk, with no eschar (Figure 7). Laboratory findings included AST 66 U/L, ALT 39 U/L, elevated CRP, and normal WBC, platelet, and neutrophil counts. CT imaging revealed no pneumonia, biliary dilation, ascites, or renal collecting system dilation.

The differential diagnosis included JSF, scrub typhus, SFTS, infectious mononucleosis, and CMV infection. Serologies for HIV, parvovirus B19, O. tsutsugamushi, measles, rubella, mumps, and EBV VCA-IgM were negative. Tetracycline therapy was initiated, leading to improvement of fever and rash. By May 7, laboratory results had improved, and she was discharged on May 8. Serology on May 17 demonstrated increased IgG titer to R. japonica, confirming JSF. She received early tetracycline therapy and recovered without complications.

We compared the clinical course and laboratory parameters from day 1 (treatment initiation) through day 3 among five patients (C1-C5). Two of the patients, C1 and C3, developed disseminated intravascular coagulation (DIC), and the disease proved fatal in C1. The timing of TC initiation varied considerably among the cases. In C1, TC was started on hospital day 9; in C2, on day 10; and in C3, on day 5. In contrast, both C4 and C5 received TC on the day of presentation. Patients with delayed initiation of TC therapy experienced longer hospital stays and developed more complications compared with those who were treated promptly.

Laboratory analyses revealed that the white blood cell (WBC) count and creatine kinase (CK) levels were significantly higher in the fatal case (C1) compared with the other patients. Specifically, WBC was markedly elevated in C1 (p = 0.023, Kruskal-Wallis test), and CK levels were also significantly increased (p = 0.041). Other laboratory parameters, including C-reactive protein (CRP), platelet count (PLT), procalcitonin (PCT), AST, ALT, and lactate dehydrogenase (LDH), did not show statistically significant differences among the cases.

When comparing baseline laboratory data across all five cases using the Kruskal-Wallis test, significant differences were observed in WBC and CK levels. Subsequently, multiple comparisons of WBC and CK values were performed using the Steel-Dwass test with Bonferroni correction. Although no significant difference was found between the fatal case (C1) and Case 2, the levels were significantly higher in C1 compared to the remaining three cases (Table 1).

**Table 1:** Baseline characteristics of five cases AST, aspartate aminotransferase; ALT, alanine aminotransferase; LDH, lactate dehydrogenase. All values in the table are presented as the median (range) for days 1 and 2 after hospitalization. *p-values were obtained using the Kruskal-Wallis test, and p < 0.005 was considered statistically significant. †p-values were obtained using the Steel-Dwass test, and p < 0.0125 (0.05/4) was considered statistically significant after Bonferroni correction.

	Case 1	Case 2	Case 3	Case 4	Case 5	p-value*
							White blood cell (/μL)	8400 (7200–9400)	6900 (6900–9900)	3700 (2900–4100)	5200 (3800–6400)	3800 (3200–4400)	0.023
p-value †		0.336	0.001	0.012	0.002	
Platelets (×10^3/μL)	97.5 (93.0-102.0)	70.5 (65-76)	114.5 (104-125)	156 (149-163)	169 (129-209)	N.S.
C-reactive protein (mg/dL)	16.97 (15.03-18.91)	18.96 (17.25-20.67)	23.18 (20.16-26.20)	4.77 (4.03-5.51)	14.775 (11.19-18.36)	N.S.
Procalcitonin (ng/mL)	0.195 (0.180-0.210)	1.355 (1.330-1.380)	-	-	1.08 (0.95-1.21)	N.S.
Creatine kinase (IU/L)	520 (372–76)	984 (220–1606)	57 (56–79)	95 (94–101)	79 (57–158)	0.041
p-value †		0.203	0.006	0.008	0.009	
AST (IU/L)	51 (47-55)	144 (140-148)	162.5 (110-215)	29 (27-31)	52 (39-65)	N.S.
ALT (IU/L)	32.5 (31-34)	81.5 (79-84)	94 (63-125)	32.5 (31-34)	33.5 (28-39)	N.S.
LDH (IU/L)	372 (363-381)	612.5 (546-679)	365 (315-415)	226 (211-241)	284.5 (253-316)	N.S.

## Discussion

We describe five JSF cases treated at our hospital from 2019-2024, including two with DIC and one death. In our small series, the fatal case (C1) demonstrated significantly elevated WBC and CK in the early treatment period. These findings, although a preliminary and exploratory observation, suggest that an elevated WBC count and CK at treatment initiation may be associated with poorer prognosis and warrant closer monitoring and more aggressive supportive care.

Prior large-scale analyses have shown that delayed initiation of tetracycline is associated with increased mortality, higher costs, and longer hospital stays. Kutsuna et al. reported higher in-hospital mortality and longer hospitalization when TC administration was delayed beyond the day of admission [10]. Likewise, Kodama et al. observed that severe complications including DIC and acute respiratory distress syndrome occurred in patients with delays of six or more days from symptom onset to initiation of therapy [11].

Our series aligns with these findings: the three patients with delayed TC initiation (C1-C3) experienced prolonged hospitalizations and greater morbidity (two DIC cases), whereas those treated with TC at presentation (C4-C5) had uncomplicated recoveries. The fatal case (C1) had notably elevated WBC and CK and demonstrated quickly progressive respiratory compromise despite later initiation of minocycline and supportive measures.

Miyashima et al. reported an association between elevated ALP and DIC risk in JSF; in their study, ALP was significantly higher in DIC patients. In our series, ALP was measured in two patients and elevated in both, one of whom developed DIC [12]. Although sample size limits conclusions, the observation is concordant with the hypothesis that hepatic involvement may predispose to coagulopathy.

The differential diagnosis of febrile rash in endemic areas should include JSF, scrub typhus, and SFTS. Laboratory clues-such as markedly elevated CRP and preserved or elevated WBC (rather than leukopenia), presence of rash including involvement of palms/soles, and eschar-may point to JSF. Early empiric treatment with tetracycline should be considered when clinical suspicion is high, given the potential for rapid deterioration.

Limitations of this report include the retrospective design and small number of cases from a single center, which limit statistical power and generalizability. Nevertheless, the concordance of our observations with larger studies supports the clinical message: early recognition and prompt tetracycline therapy are critical to prevent severe outcomes in JSF.

## Conclusions

In this case series of five JSF patients, elevated WBC and CK during the early treatment phase were observed in the fatal case and may serve as markers of severe disease. Delayed initiation of TC was linked to worse clinical courses, including DIC and prolonged hospitalization. Prompt clinical suspicion, early empiric therapy with tetracycline, and close monitoring of laboratory markers (WBC and CK) are recommended to reduce the risk of severe outcomes.
